# Expression Atlas in 2026: enabling FAIR and open expression data through community collaboration and integration

**DOI:** 10.1093/nar/gkaf1238

**Published:** 2025-12-10

**Authors:** Pedro Madrigal, Anil S Thanki, Silvie Fexova, Iris D Yu, Arsenios Chatzigeorgiou, Ida Zucchi, Jose C Marugan Calles, Liora Vilmovsky, Amnon Khen, Lingyun Zhao, Karoly Erdos, Sandeep R Kurri, Sandeep Selvakumar, Upendra Kumbham, Ananth Prakash, Shengbo Wang, Andrew Green, Carlos Eduardo Ribas, Blake Sweeney, Tobi Alegbe, Daniel Suveges, Anmol Hemrom, David E Gomez Gutierrez, Santiago Insua, Matt Jeffryes, Matt Pearce, Prasad Basutkar, Myrsini Kaforou, Aubrey Cunnington, Michael Levin, Sunita Kumari, Doreen Ware, Damien Goutte-Gattat, Katja Röper, Nicholas H Brown, Yanhui Hu, Norbert Perrimon, Irene Papatheodorou, Alvis Brazma, Henning Hermjakob, Melissa Harrison, David Ocaña, David Ochoa, Ellen M McDonagh, Alex Bateman, Thomas Keane, Juan Antonio Vizcaíno, Christina Ernst

**Affiliations:** European Molecular Biology Laboratory, European Bioinformatics Institute (EMBL-EBI), Wellcome Genome Campus, Hinxton CB10 1SD, United Kingdom; European Molecular Biology Laboratory, European Bioinformatics Institute (EMBL-EBI), Wellcome Genome Campus, Hinxton CB10 1SD, United Kingdom; European Molecular Biology Laboratory, European Bioinformatics Institute (EMBL-EBI), Wellcome Genome Campus, Hinxton CB10 1SD, United Kingdom; European Molecular Biology Laboratory, European Bioinformatics Institute (EMBL-EBI), Wellcome Genome Campus, Hinxton CB10 1SD, United Kingdom; European Molecular Biology Laboratory, European Bioinformatics Institute (EMBL-EBI), Wellcome Genome Campus, Hinxton CB10 1SD, United Kingdom; European Molecular Biology Laboratory, European Bioinformatics Institute (EMBL-EBI), Wellcome Genome Campus, Hinxton CB10 1SD, United Kingdom; European Molecular Biology Laboratory, European Bioinformatics Institute (EMBL-EBI), Wellcome Genome Campus, Hinxton CB10 1SD, United Kingdom; European Molecular Biology Laboratory, European Bioinformatics Institute (EMBL-EBI), Wellcome Genome Campus, Hinxton CB10 1SD, United Kingdom; European Molecular Biology Laboratory, European Bioinformatics Institute (EMBL-EBI), Wellcome Genome Campus, Hinxton CB10 1SD, United Kingdom; European Molecular Biology Laboratory, European Bioinformatics Institute (EMBL-EBI), Wellcome Genome Campus, Hinxton CB10 1SD, United Kingdom; European Molecular Biology Laboratory, European Bioinformatics Institute (EMBL-EBI), Wellcome Genome Campus, Hinxton CB10 1SD, United Kingdom; European Molecular Biology Laboratory, European Bioinformatics Institute (EMBL-EBI), Wellcome Genome Campus, Hinxton CB10 1SD, United Kingdom; European Molecular Biology Laboratory, European Bioinformatics Institute (EMBL-EBI), Wellcome Genome Campus, Hinxton CB10 1SD, United Kingdom; European Molecular Biology Laboratory, European Bioinformatics Institute (EMBL-EBI), Wellcome Genome Campus, Hinxton CB10 1SD, United Kingdom; European Molecular Biology Laboratory, European Bioinformatics Institute (EMBL-EBI), Wellcome Genome Campus, Hinxton CB10 1SD, United Kingdom; European Molecular Biology Laboratory, European Bioinformatics Institute (EMBL-EBI), Wellcome Genome Campus, Hinxton CB10 1SD, United Kingdom; European Molecular Biology Laboratory, European Bioinformatics Institute (EMBL-EBI), Wellcome Genome Campus, Hinxton CB10 1SD, United Kingdom; European Molecular Biology Laboratory, European Bioinformatics Institute (EMBL-EBI), Wellcome Genome Campus, Hinxton CB10 1SD, United Kingdom; European Molecular Biology Laboratory, European Bioinformatics Institute (EMBL-EBI), Wellcome Genome Campus, Hinxton CB10 1SD, United Kingdom; European Molecular Biology Laboratory, European Bioinformatics Institute (EMBL-EBI), Wellcome Genome Campus, Hinxton CB10 1SD, United Kingdom; OpenTargets, EMBL-EBI, Wellcome Genome Campus, Hinxton CB10 1SD, United Kingdom; European Molecular Biology Laboratory, European Bioinformatics Institute (EMBL-EBI), Wellcome Genome Campus, Hinxton CB10 1SD, United Kingdom; OpenTargets, EMBL-EBI, Wellcome Genome Campus, Hinxton CB10 1SD, United Kingdom; European Molecular Biology Laboratory, European Bioinformatics Institute (EMBL-EBI), Wellcome Genome Campus, Hinxton CB10 1SD, United Kingdom; European Molecular Biology Laboratory, European Bioinformatics Institute (EMBL-EBI), Wellcome Genome Campus, Hinxton CB10 1SD, United Kingdom; European Molecular Biology Laboratory, European Bioinformatics Institute (EMBL-EBI), Wellcome Genome Campus, Hinxton CB10 1SD, United Kingdom; European Molecular Biology Laboratory, European Bioinformatics Institute (EMBL-EBI), Wellcome Genome Campus, Hinxton CB10 1SD, United Kingdom; European Molecular Biology Laboratory, European Bioinformatics Institute (EMBL-EBI), Wellcome Genome Campus, Hinxton CB10 1SD, United Kingdom; European Molecular Biology Laboratory, European Bioinformatics Institute (EMBL-EBI), Wellcome Genome Campus, Hinxton CB10 1SD, United Kingdom; Section of Paediatric Infectious Disease, Department of Infectious Disease, and Centre for Paediatrics and Child Health, Imperial College London, London SW7 2AZ, United Kingdom; Section of Paediatric Infectious Disease, Department of Infectious Disease, and Centre for Paediatrics and Child Health, Imperial College London, London SW7 2AZ, United Kingdom; Section of Paediatric Infectious Disease, Department of Infectious Disease, and Centre for Paediatrics and Child Health, Imperial College London, London SW7 2AZ, United Kingdom; Cold Spring Harbor Laboratory, 1 Bungtown Road, Cold Spring Harbor, NY 11724, United States; Cold Spring Harbor Laboratory, 1 Bungtown Road, Cold Spring Harbor, NY 11724, United States; FlyBase-Cambridge, Department of Physiology, Development and Neuroscience, University of Cambridge, Downing Street, Cambridge CB2 3DY, United Kingdom; FlyBase-Cambridge, Department of Physiology, Development and Neuroscience, University of Cambridge, Downing Street, Cambridge CB2 3DY, United Kingdom; FlyBase-Cambridge, Department of Physiology, Development and Neuroscience, University of Cambridge, Downing Street, Cambridge CB2 3DY, United Kingdom; Department of Genetics, Harvard Medical School, Boston, MA 02115, United States; Department of Genetics, Harvard Medical School, Boston, MA 02115, United States; European Molecular Biology Laboratory, European Bioinformatics Institute (EMBL-EBI), Wellcome Genome Campus, Hinxton CB10 1SD, United Kingdom; European Molecular Biology Laboratory, European Bioinformatics Institute (EMBL-EBI), Wellcome Genome Campus, Hinxton CB10 1SD, United Kingdom; European Molecular Biology Laboratory, European Bioinformatics Institute (EMBL-EBI), Wellcome Genome Campus, Hinxton CB10 1SD, United Kingdom; European Molecular Biology Laboratory, European Bioinformatics Institute (EMBL-EBI), Wellcome Genome Campus, Hinxton CB10 1SD, United Kingdom; European Molecular Biology Laboratory, European Bioinformatics Institute (EMBL-EBI), Wellcome Genome Campus, Hinxton CB10 1SD, United Kingdom; European Molecular Biology Laboratory, European Bioinformatics Institute (EMBL-EBI), Wellcome Genome Campus, Hinxton CB10 1SD, United Kingdom; OpenTargets, EMBL-EBI, Wellcome Genome Campus, Hinxton CB10 1SD, United Kingdom; European Molecular Biology Laboratory, European Bioinformatics Institute (EMBL-EBI), Wellcome Genome Campus, Hinxton CB10 1SD, United Kingdom; OpenTargets, EMBL-EBI, Wellcome Genome Campus, Hinxton CB10 1SD, United Kingdom; Wellcome Trust Sanger Institute, Wellcome Genome Campus, Hinxton CB10 1SD, United Kingdom; European Molecular Biology Laboratory, European Bioinformatics Institute (EMBL-EBI), Wellcome Genome Campus, Hinxton CB10 1SD, United Kingdom; European Molecular Biology Laboratory, European Bioinformatics Institute (EMBL-EBI), Wellcome Genome Campus, Hinxton CB10 1SD, United Kingdom; European Molecular Biology Laboratory, European Bioinformatics Institute (EMBL-EBI), Wellcome Genome Campus, Hinxton CB10 1SD, United Kingdom; European Molecular Biology Laboratory, European Bioinformatics Institute (EMBL-EBI), Wellcome Genome Campus, Hinxton CB10 1SD, United Kingdom

## Abstract

Expression Atlas (https://www.ebi.ac.uk/gxa/home) is EMBL-EBI’s comprehensive knowledgebase for gene and protein expression across tissues, cell types, conditions, and multiple species. Since our last update, Expression Atlas has expanded substantially in both content and functionality, now comprising >4500 studies from 67 species, with increased proteomics coverage and updated Genotype-Tissue Expression (GTEx) tissue profiles. The resource also includes hundreds of single-cell RNA-seq experiments spanning 21 species, among them externally analysed community datasets such as Tabula Sapiens and GTEx single-nucleus profiles, allowing exploration of curated atlases while maintaining their original analytical framework. Key methodological advances include a new marker gene analysis module for bulk baseline experiments, alongside workflow updates that improve reproducibility. Expression Atlas data are integrated into EMBL-EBI resources such as Ensembl, UniProt, and Europe PMC and disseminated through collaboration with model organism communities such as FlyBase and Gramene. The resource also supports translational research through the European Diagnostic Transcriptomic Library and integration with the Open Targets platform. Future directions include modernizing analysis pipelines, enhancing programmatic access, and delivering AI-ready data formats, strengthening Expression Atlas as a findable, accessible, interoperable, and reusable (FAIR) community-driven resource for both fundamental and translational discovery.

## Graphical Abstract

**Graphical Abstract:**
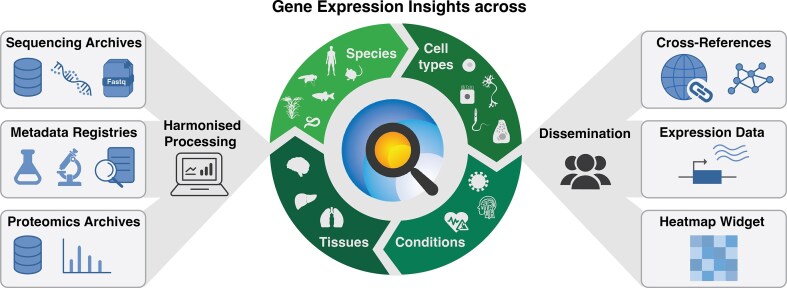

## Introduction

Understanding where and under what conditions genes are expressed is fundamental to biology and medicine. Expression Atlas (https://www.ebi.ac.uk/gxa/home) was established in 2009 as an added-value knowledgebase to enable researchers to query gene and protein expression patterns across tissues, cell types, conditions, and multiple species, including human, model, and non-model organisms [1]. It aggregates high-quality transcriptomics and proteomics datasets, re-analysed through standardized pipelines and presented in an accessible, integrated manner, in agreement with the principles of making data findable, accessible, interoperable, and reusable (FAIR) [2]. Transcriptomic datasets are primarily sourced from functional genomics archives such as ArrayExpress [3] and Gene Expression Omnibus (GEO) [4], including selected studies under managed access in the European Genome-Phenome Archive (EGA) [5] or NCBI’s Database of Genotypes and Phenotypes (dbGaP) [6], while proteomics datasets are obtained through a close collaboration with EMBL-EBI’s PRoteomics IDEntifications (PRIDE) database [7].

Initially, Expression Atlas featured two main study types: baseline experiments reporting gene expression in normal (untreated) conditions across tissues and differential experiments reporting changes in expression under various perturbations, including diseases, treatments, genetic modifications, etc. Over the past decade, these have grown to cover a wide taxonomic breadth, now hosting 67 species and multiple data types, including microarrays, bulk RNA sequencing, and mass spectrometry (MS)-based proteomics.

In recent years, with the surge of single-cell genomics and transcriptomics technologies, Expression Atlas was extended with functionality to incorporate single-cell gene expression data. Since 2018, this single-cell component of Expression Atlas [8] has provided dedicated support for single-cell RNA sequencing (scRNA-seq) studies. Together, the bulk and single-cell views within Expression Atlas enable users to investigate when and where genes are expressed, whether in normal tissue contexts or in response to specific conditions.

This article provides an update on Expression Atlas since the last NAR (*Nucleic Acids Research*) Database Issue Report in 2024 [9]. We summarize current content and statistics, describe new data types and species incorporated, and highlight improvements in analysis pipelines and user features. We also detail ongoing community collaborations and data dissemination efforts that extend the Atlas’ reach, and outline future plans aimed at ensuring Expression Atlas data are readily usable for machine learning approaches.

## Data growth and content

### Expression atlas statistics

At the time of writing, the latest release of Expression Atlas (release 43, 2025) contains 4562 studies from 67 species, representing substantial growth since our 2024 report [9]. These encompass ~2900 legacy microarray studies, 1512 RNA-seq experiments, and 123 proteomics studies, adding up to >160 000 assays. The baseline layer now covers 375 experiments across 48 organisms, while the differential layer comprises 4187 experiments across 67 organisms (Table 1). This makes Expression Atlas one of the largest uniformly re-analysed expression resources globally, with unique breadth across humans and both model and non-model organisms.

**Table 1.** Top 10 species represented in Expression Atlas, ranked by the number of studies

Species	Number of differential studies	Number of baseline studies
*Homo sapiens*	1528	123
*Mus musculus*	1239	65
*Arabidopsis thaliana*	615	18
*Rattus norvegicus*	172	14
*Drosophila melanogaster*	145	5
*Oryza sativa*	98	15
*Zea mays*	58	33
*Saccharomyces cerevisiae*	49	2
*Gallus gallus*	35	4
*Caenorhabditis elegans*	32	1

In addition to these species, Expression Atlas includes 216 differential and 95 baseline studies across additional species.

### Taxonomic expansion: first protist species in Expression Atlas

The latest Expression Atlas release includes *Dictyostelium discoideum*, our first protist species, increasing the total number of represented organisms in the knowledge base to 67. The incorporated study examines terminally differentiated cells (spores, stalk, and cup cells) to provide insights into the ancestry and evolution of novel somatic cell types in slime moulds and serves as a resource for investigating multicellularity [10]. The inclusion of *Dictyostelium* highlights our ongoing commitment to cover evolutionary diversity.

### Proteomics data

Since the last update, we have expanded the proteomics content of Expression Atlas, in collaboration with the PRIDE team at EMBL-EBI, who provide uniformly re-analysed MS-based datasets [7]. Coverage has increased from 93 to 123 studies since 2024, including 119 baseline and 4 differential proteomics datasets. Most datasets come from healthy human and model organism tissues, as well as cancer cell line samples [11, 12].

New proteomics experiments include comprehensive baseline protein profiles from healthy pig tissues using data-dependent acquisition (DDA) and from human tissues using data-independent acquisition (DIA). Furthermore, cross-links have been introduced between related transcriptomic and proteomic experiments, supporting integration across modalities [13]. The pilot collection of DIA experiments already in Expression Atlas [14], which covered cell lines, plasma, and human cancer samples, has been expanded with 15 additional public studies profiling baseline protein abundances across a range of healthy tissue samples [15]. Updates to the DIA data re-analysis pipeline included processing data with DIA-NN v1.8.1 [16] using an *in silico* entrapment spectral library [15]; these datasets are tagged ‘Human2024_DIA.’

In addition to the human datasets, we have incorporated the *Arabidopsis* proteomic tissue atlas [17] and 14 new DDA baseline studies from pigs [18], including a large-scale dataset spanning nine tissues [19] and additional studies of the gastrointestinal tract, heart, skeletal muscle, liver, adipose tissue, and retina. Together, these additions underscore the growing importance of proteomics in Expression Atlas and highlight our commitment to supporting multi-omics integration.

### GTEx integration and updates

**Figure 1.**
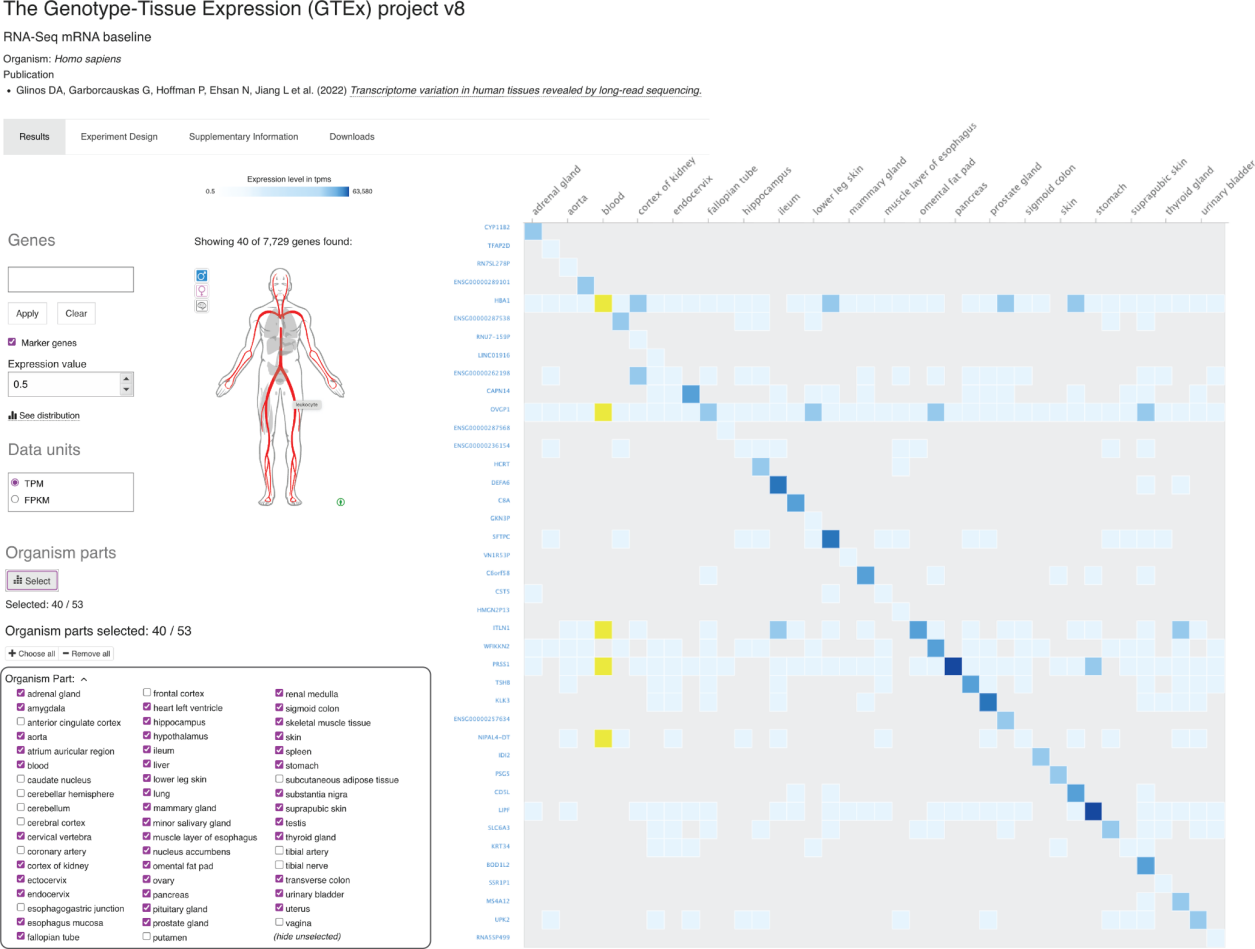
Heatmap visualization of GTEx V8 expression profiles showing the most specific marker genes across 40 selected human tissues (https://www.ebi.ac.uk/gxa/experiments/E-GTEX-8/). This view highlights tissue-restricted expression patterns and is representative of the new marker gene module implemented for all transcriptomic and proteomic baseline studies.

One of the largest individual contributions to Expression Atlas is the Genotype-Tissue Expression (GTEx) project. We have updated to GTEx release 8 (V8), which comprises ~17 300 RNA-seq samples collected from 54 distinct tissue sites from ~948 post-mortem donors [20]. This provides near-comprehensive coverage of human tissues with improved quality control, underpinning cross-study comparisons and downstream analyses [21]. In Expression Atlas, users can explore GTEx expression profiles for their genes of interest, examine tissue specificity by selecting specific marker genes, and download normalized counts for re-analysis (Fig. 1). By integrating GTEx alongside many other human studies, Expression Atlas enables users to assess whether a gene is broadly expressed, tissue-restricted, or altered under specific conditions and diseases. This integration broadens the impact of GTEx by situating its data within a wider experimental landscape, making it possible to move seamlessly from baseline tissue expression to differential expression across diverse contexts.

### Single-cell expression atlas

The latest release of Single Cell Expression Atlas (SCEA; release 21, 2024) contains a total of 383 single-cell RNA-seq experiments, comprising >10 million cells, across 21 species. The most represented organisms remain human and mouse, with growing contributions from model species such as *Drosophila* and *Arabidopsis* (Table 2). Notable additions include the Aging Fly Cell Atlas [22] and a dataset of ∼850 000 cells from the developing *Drosophila* optic lobes, mapping transcriptional programmes of visual circuit assembly [23]. Release 21 also introduced a new interactive human gut anatomogram that provides a zoomable anatomical map linked to cell-level data, enabling users to navigate from tissue-scale views to specific cell-type heatmaps within the same interface.

**Table 2.** Top 8 species represented in Single Cell Expression Atlas, ranked by the number of studies

Species	Number of studies
*Homo sapiens*	159
*Mus musculus*	125
*Drosophila melanogaster*	41
*Danio rerio*	15
*Arabidopsis thaliana*	14
*Gallus gallus*	4
*Rattus norvegicus*	3
*Oryza sativa*	3

### Ingestion of externally analysed data

**Figure 2.**
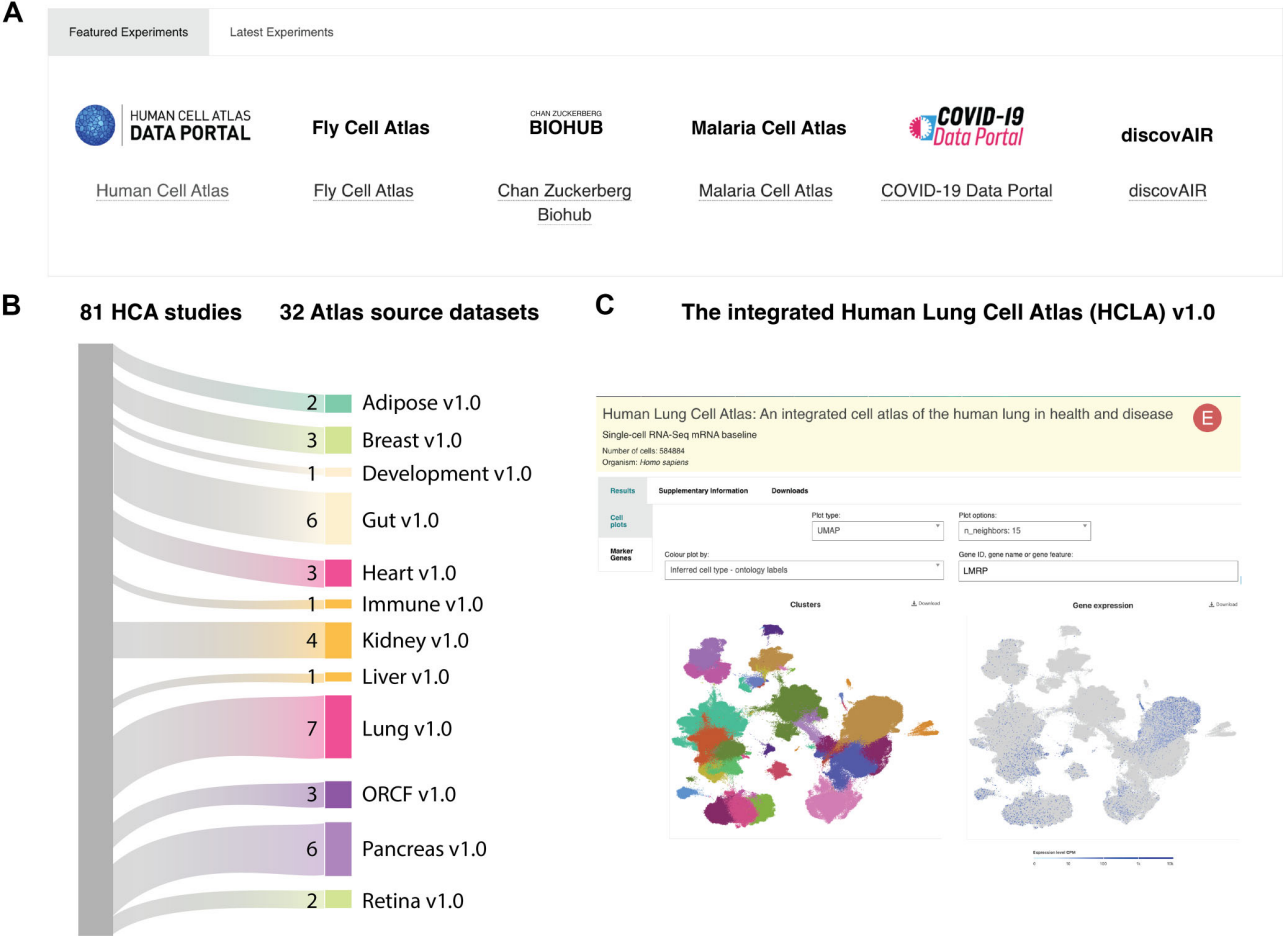
The Human Cell Atlas–Data Portal collection in SCEA contains data from 81 Human Cell Atlas (HCA) studies, out of which 32 are source datasets for 12 upcoming or existing integrated atlases. Of note, some studies are used across many atlases. (**A**) Screenshot of the SCEA web application panel (https://www.ebi.ac.uk/gxa/sc/) displaying the HCA collection of experiments alongside other featured collections such as the Fly Cell Atlas and COVID-19 Data Portal [33]. (**B**) Sankey plot showing the shared studies between HCA and SCEA and the distribution of source datasets across atlases. (**C**) Example experiment page for the Human Lung Cell Atlas in SCEA, the first externally analysed HCA dataset included (https://www.ebi.ac.uk/gxa/sc/experiments/E-ANND-1/). These views illustrate how SCEA can integrate community atlases while preserving their original analyses.

In addition to our in-house processing pipelines, the latest SCEA release introduces a new class of externally analysed data, ingested as pre-processed AnnData objects [24] and assigned accession IDs in the format E-ANND-X. Selected experiments from GTEx, Tabula Sapiens, the Human Lung Cell Atlas, and the Developing Human Immune System Atlas were included and marked with an ‘E’ icon (Fig. 2C). This approach enables SCEA to integrate large, high-quality single-cell atlases without duplicating computation and acknowledging the analytical choices of the original consortia.

The GTEx single-nucleus RNA-seq atlas profiles >200 000 nuclei from 16 donors across 25 human tissues, providing a comprehensive cross-tissue reference for healthy cellular composition [25]. For Tabula Sapiens, we include only the high-coverage Smart-seq2 dataset (∼500 000 cells from 24 human tissues and 475 annotated cell types), which offers exceptional resolution [26]. The Human Lung Cell Atlas integrates healthy and diseased lung samples, providing insights into pulmonary cell diversity and pathology [27]. The Developing Human Immune System Atlas provides temporal profiles of the immune system development from fetal to adult stages [28].

### Human Cell Atlas collection in SCEA

The datasets mentioned earlier are all part of, or have been used in, the HCA project [29]. Their addition brings the total number of HCA datasets in SCEA to 81, represented as 94 experiments and highlighted as a featured collection in the experiments browser (Fig. 2A). This collection includes integrated atlases, underlying source studies, and other HCA-ingested datasets (Fig. 2B). Comprehensive information about these datasets can be obtained through the HCA Data Portal (https://data.humancellatlas.org/).

By supporting the ingestion of externally analysed datasets in AnnData format, SCEA preserves author-driven analytical decisions, such as batch correction and cell-type annotation, while enabling users to explore gene expression, marker genes, and metadata of high interest for the single-cell community within our interface. This approach also represents an important first step towards making SCEA more interoperable with other community resources such as Bgee [30] or CZ CELLxGENE [31]. We plan to expand this mode of data ingestion to additional community atlases and to formalize provenance tracking, e.g. by following matrix and analysis metadata standards [32].

## Methodology, analysis workflow, and infrastructure improvements

### Marker gene identification for Expression Atlas baseline experiments

Understanding which genes are most specific to a given tissue or condition is key for interpreting baseline expression data. To support this, we added a new analysis module that identifies marker genes for each experimental group. Marker Gene Finder in RNA-seq data (MGFR) computes whether the highest expression for a gene occurs exclusively in one tissue or condition group and assigns a specificity score between 0 and 1, where lower values (closer to 0) indicate higher specificity and values nearer 1 indicate broad expression [34]. In the current implementation, marker gene lists are defined as having a specificity score <0.3 and expression level >0.5 TPMs (transcripts per million) and are available from our FTP site (https://ftp.ebi.ac.uk/pub/databases/microarray/data/atlas/experiments/).

By selecting the ‘Marker genes’ option in the interface of a baseline experiment, the heatmap displays expression levels for the top marker genes, with the current release showing marker genes across all selected conditions (Fig. 1). Future improvements to this feature will include additional filtering options based on biotype (e.g. protein-coding, lncRNA) and controls for adjusting the number of marker genes shown per condition.

### End-to-end Nextflow-based single-cell workflow

For SCEA we completed the transition of the remaining Galaxy-based components to Nextflow, providing an end-to-end workflow with improved scalability, reproducibility, and containerization. The pipeline employs technology-specific quantifiers (e.g. Alevin/Salmon for droplet-based libraries) and includes updated quality control, doublet detection, and batch-correction steps. Our downstream analysis remains based on Scanpy [35] and follows nf-core community standards where possible [36, 37].

Following the move to Nextflow, our pipeline was further refined during the March 2025 nf-core hackathon (https://nf-co.re/events/2025/hackathon-march-2025.html), where our team actively participated alongside the broader community to update specific components of our SCEA workflows. Key improvements include automated testing, updated clustering algorithms [38], and the adoption of semantic versioning. Together these changes enhance reproducibility and sustainability, making the workflow broadly usable beyond our team while aligning with community best practices.

### Infrastructure and deployment modernization

Alongside data analysis and interface developments, we are modernizing the underlying infrastructure by migrating to a fully containerized application stack orchestrated with Kubernetes. This shift improves scalability, fault tolerance, and maintainability, enabling us to handle growing dataset volumes and user traffic while simplifying deployment of new features and services. Containerization and Kubernetes orchestration ensure a more reliable service, with automatic scaling to meet demand, efficient resource use, and zero-downtime updates.

## Data dissemination and community collaboration

### Data exports and integration with other EMBL-EBI resources

Expression Atlas data are disseminated beyond the web interface through regular exports to other EMBL-EBI services [39] and external partners, ensuring that expression information is findable and accessible across multiple entry points. These integrations not only provide interoperability but also enrich the recipient resources, enabling them to place expression data in a cell, organ, or tissue context.

**Figure 3.**
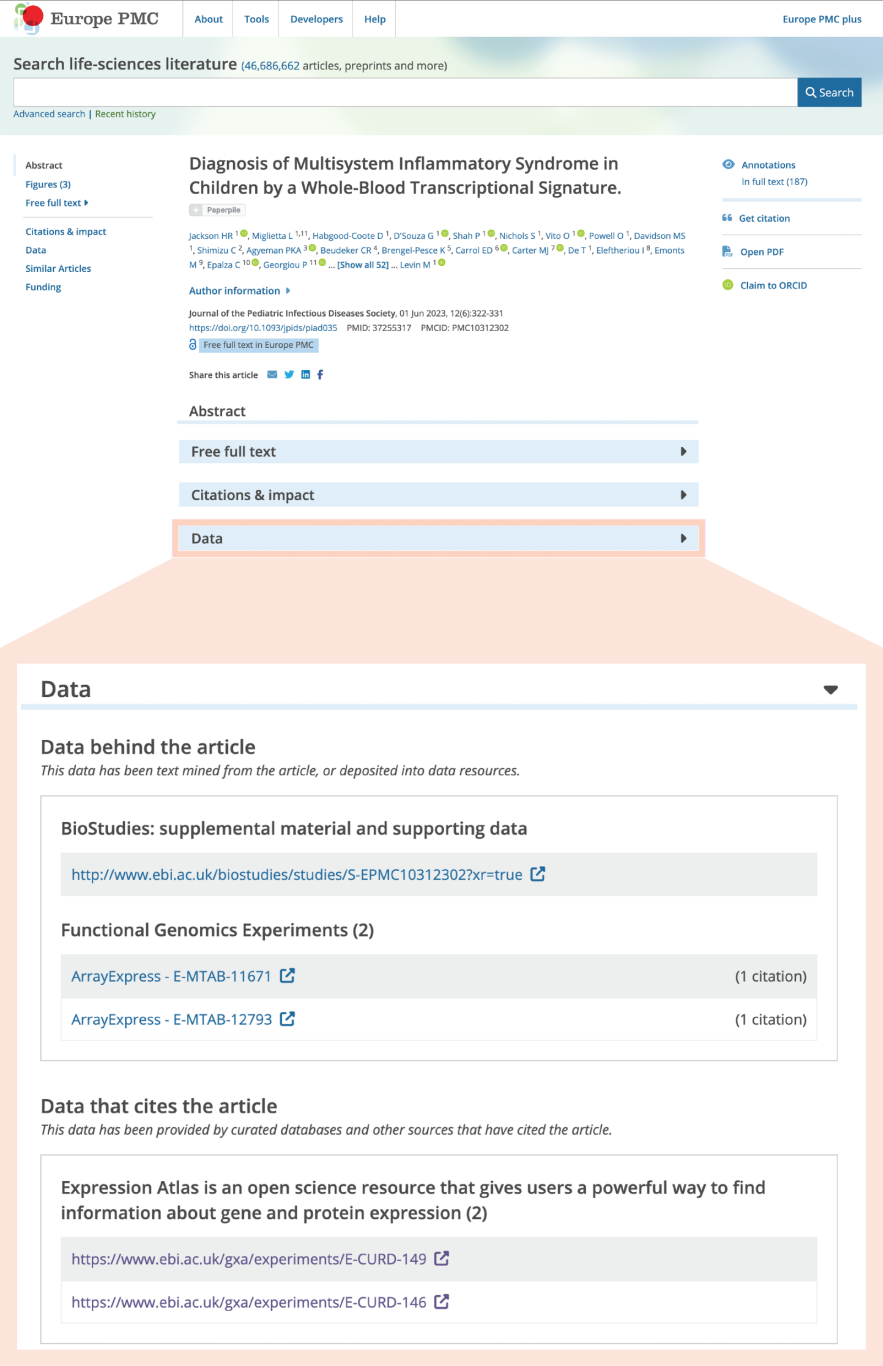
Example of Europe PMC web portal displaying embedded links to Expression Atlas datasets from the European Diagnostic Transcriptomic Library (EDTL) (https://europepmc.org/article/MED/37255317). Cross-references point to the corresponding RNA-seq data, visualized in Expression Atlas and archived in the ArrayExpress collection in BioStudies. The database links component of the Europe PMC record is accessible through the Europe PMC API at https://www.ebi.ac.uk/europepmc/webservices/rest/MED/37255317/datalinks.

Exports are used to generate direct cross-references in other databases, for instance UniProt [40] (https://www.uniprot.org/database/DB-0004), enabling users to connect protein function with gene expression. We have also recently established links with Europe PMC [41], embedding Expression Atlas datasets within publication records alongside existing data links, so that literature searches can lead directly to the underlying expression data (Fig. 3).

Beyond cross-references, Atlas datasets are indexed in EBI Search, a scalable text-search engine that provides uniform access to EMBL-EBI resources [42]. Five dedicated domains—atlas-experiments, atlas-genes, atlas-genes-differential, sc-experiments, and sc-genes—enable targeted retrieval of baseline, differential, and single-cell data. Our export pipeline indexes data into EBI Search, thus providing a unified metadata view for user queries (https://www.ebi.ac.uk/ebisearch/).

**Figure 4.**
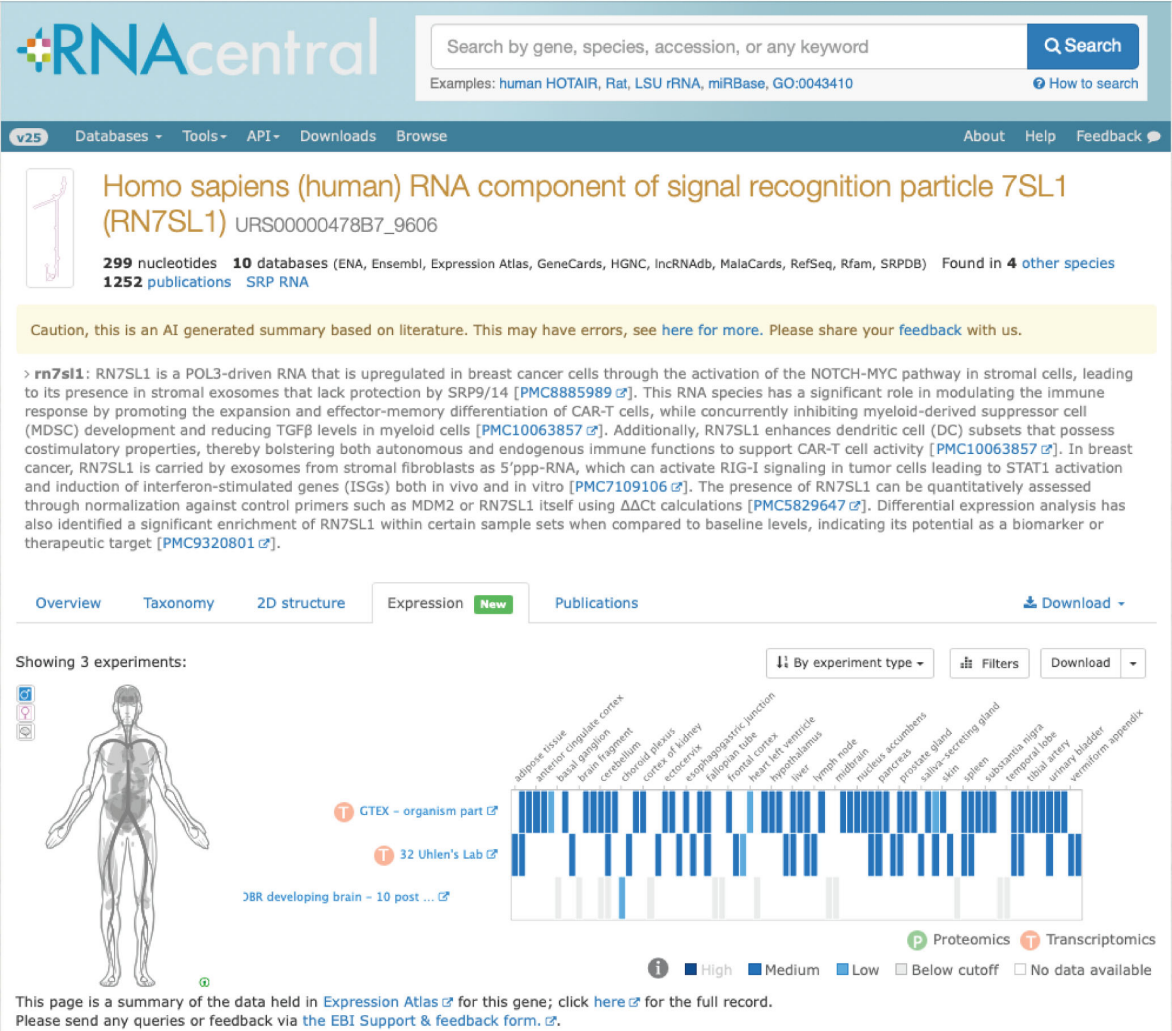
RNAcentral gene page showing the Expression Atlas heatmap widget for human non-coding RNA *RN7SL1* (https://rnacentral.org/rna/URS00000478B7/9606).

Finally, Expression Atlas data can be accessed through our embeddable heatmap widget. This visualization is integrated into multiple EMBL-EBI resources such as Ensembl [43] and RNAcentral, providing contextual expression information directly on gene pages. The widget code and integration instructions are openly available on our GitHub repository (https://github.com/ebi-gene-expression-group/atlas-heatmap), allowing any resource to incorporate Atlas visualizations, and have already been adopted by external databases such as Gramene. In RNAcentral, for instance, the widget highlights relevant studies and samples where a given non-coding RNA is expressed (Fig. 4), with cross-links back to the corresponding Atlas experiment for further exploration [44].

### Global consortia and model organism community collaborations

We continue to collaborate with major consortia and model organism communities such as the Human Cell Atlas, FlyBase [45], and Gramene [46] to ingest, analyse, and share expression data. These collaborations span both upstream and downstream interactions: some partners help prioritize and curate datasets for inclusion in Expression Atlas, while others integrate Atlas outputs to enrich their own knowledge bases.

Our collaboration with Gramene focuses on plant genomics, with Gramene serving as a key partner for identifying and prioritizing plant datasets for inclusion in Expression Atlas. Currently, 1026 plant studies from 27 species constitute >20% of our total collection, representing one of the largest collections of plant transcriptomic studies available through a single resource (Supplementary Tables S1 and S2). Gramene provides expert curation support for crop species and helps ensure agricultural research communities can easily access relevant expression data across diverse plant species and conditions.

Our partnership with FlyBase enables seamless integration of *Drosophila* expression data, where FlyBase assists with curation of datasets, incorporates our processed single-cell datasets, and provides enhanced gene expression summaries to their users. The collaboration ensures that fly researchers have access to both individual study results in Expression Atlas and cross-study comparisons through FlyBase’s familiar interface.

Other downstream partners include the Mouse Gene Expression Database (GXD) at MGI [47] and the Rat Genome Database (RGD) [48], which import Atlas data to strengthen their communities’ access to standardized transcriptomic information. For GXD specifically, TPM values are loaded from Expression Atlas on demand as new relevant experiments become available, enabling integration of RNA-seq with classical expression data—such as RNA *in situ* hybridization or northern blot—using consistent present/absent calls derived from Atlas TPM ranges.

### Clinical transcriptomics resource for diagnostics

The European Diagnostic Transcriptomic Library (EDTL) aims to build reference panels for a molecular taxonomy of infectious and inflammatory diseases, which can be harnessed for rapid transcriptomic diagnostics. As partners in the DIAMONDS consortium (https://www.diamonds2020.eu/) that brings together clinicians, researchers, and computational biologists, we have added two initial datasets to the EDTL collection (https://www.ebi.ac.uk/gxa/edtl/experiments). Expression Atlas provides curated data and analysis infrastructure for the DIAMONDS consortium, ensuring that datasets are findable and explorable by the broader research community. Current data captures a diverse range of well-characterized infectious (e.g. malaria, tuberculosis, meningococcal disease, and influenza) and inflammatory diseases (e.g. Kawasaki disease, juvenile idiopathic arthritis, and multisystem inflammatory disease in children) [49, 50]. The consortium plans to add further data from thousands more subjects as the project progresses.

### Use of expression atlas in drug discovery

Open Targets (OT) is a partnership between EMBL-EBI, the Wellcome Sanger Institute, and five pharmaceutical companies, with the aim of identifying and prioritizing targets for developing safer and more effective drugs [51]. OT collaborates with Expression Atlas through a meta-analysis of over 18 000 samples from 50 different tissues and >30 cell types (https://platform-docs.opentargets.org/target/baseline-expression). This meta-analysis uses Expression Atlas baseline exports for RNA expression to assess whether a target is expressed in all tissues or selectively in specific tissues or cell types. The availability of target molecules in relevant locations is critical at different stages of the drug development process. Expression data can help in understanding which tissues and cell types are relevant in disease, which genes are differentially expressed in disease, and evaluation of specificity and distribution to understand how safe it may be to modulate a target and where to modulate it in the body for therapeutic effect.

**Figure 5.**
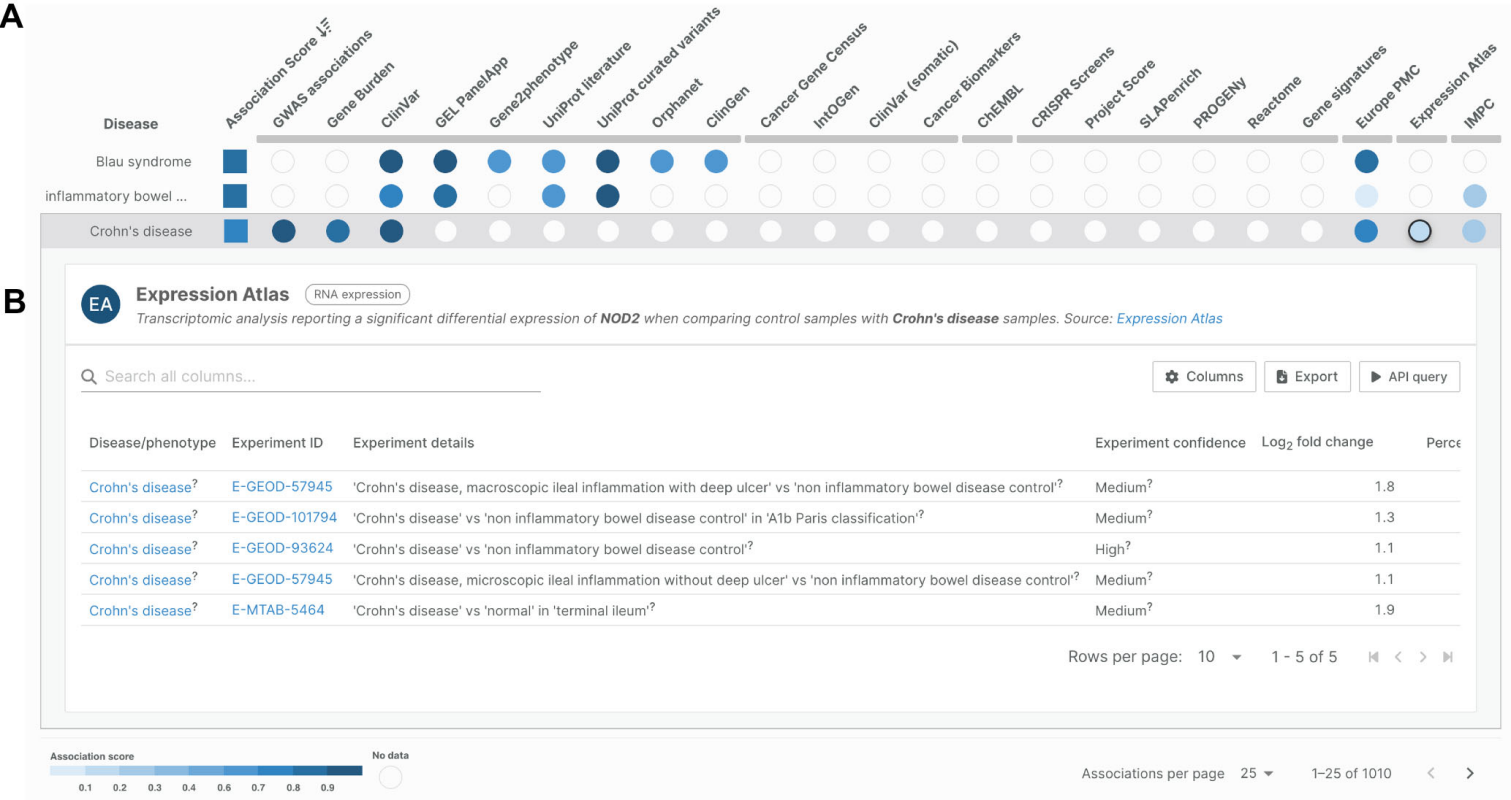
Expression Atlas differential expression evidence in the OTs Platform for human gene *NOD2*, a gene implicated in the development and pathogenesis of Crohn’s disease. (**A**) Availability of expression data visualized as a coloured circle. (**B**) Expanded panel displaying details of the underlying studies with direct links to Expression Atlas (https://platform.opentargets.org/target/ENSG00000167207/associations).

In the OT Platform (https://platform.opentargets.org/), each contrast from independent studies capturing differentially regulated genes constitutes independent evidence. OT builds an Expression Atlas evidence score for target-disease associations, which takes into account three measures: scaled *P*-value from 0 (*P* = 1) to 1 (*P *< 1e^−10^), absolute log_2_ fold change divided by 10, and percentile rank divided by 100 (Fig. 5). Expression Atlas weights are low because gene expression is ranked below other evidence types for disease association; for example, GWAS associations are more likely to indicate causal variants, whereas expression changes may instead reflect downstream effects of disease progression. Future development in the OT Platform will integrate RNA expression from Atlas in a comparative widget for different tissues offered to the user to investigate disease associations on the fly [52].

## Future directions

### Community data submissions and engagement

We are developing enhanced mechanisms for community data submissions and engagement, building on our existing collaborations with model organism databases and clinical research consortia. These efforts will focus on streamlining the submission process while maintaining our high curation standards.

### Modernized analysis pipeline for expression atlas

A modernized analysis pipeline for bulk Expression Atlas is in development to replace the existing iRAP workflow [53]. The new pipeline will be designed using modern workflow managers and will be modular and easily adaptable by the wider community. This redesign will improve computational efficiency, decrease computing carbon footprint, enhance reproducibility, facilitate scalability, and enable easier integration with other bioinformatics tools and platforms.

### Meta-analysis dataset integration for bulk data

A new analysis module is under development for Expression Atlas that will introduce a view summarizing gene expression across selected key tissues or organs by integrating data from multiple studies from an organism. It will include baseline experiments combined using meta-analysis and batch-correction methods, enabling researchers to obtain more robust expression estimates by leveraging data from multiple high-quality studies.

### Enhanced Bioconductor package

The current version of the ExpressionAtlas R package (v2.0.0) (https://bioconductor.org/packages/ExpressionAtlas) enables users to search and download microarray and bulk RNA-seq data from Expression Atlas. We are developing an enhanced version with capabilities for searching both bulk and single-cell expression atlas studies via EBI RESTful Web Services and the Expression Atlas API. The updated package will include visualization functionality to plot heatmaps and clusters, making it easier for R users to integrate Expression Atlas data into their analysis workflows and to customize plots.

### AI-ready data formats for ML applications

We are planning to update how we serve data by adopting machine learning and artificial intelligence-friendly formats for expression data. This initiative will enable Expression Atlas to serve as a robust source for model training applications and foundation models, with our wide taxonomic range across 67 species supporting the development of more comprehensive models of gene expression. The planned enhancements include optimized data structures, standardized feature representations, and batch download capabilities specifically designed for computational approaches. These developments will support the growing intersection of genomics and AI, allowing researchers to leverage Expression Atlas data for training predictive models, developing new analytical methods, and advancing computational biology applications.

## Supplementary Material

gkaf1238_Supplemental_File

## Data Availability

Expression Atlas and SCEA are available at https://www.ebi.ac.uk/gxa and https://www.ebi.ac.uk/gxa/sc, respectively. The Expression Atlas web applications and data analysis pipelines are open source and maintained within the GitHub organization of the Gene Expression Group at EMBL-EBI. The Expression Atlas web application is available via https://github.com/ebi-gene-expression-group/atlas-web-single-cell (DOI: 10.5281/zenodo.10021405) for single-cell data and https://github.com/ebi-gene-expression-group/atlas-web-bulk (DOI: 10.5281/zenodo.10021637) for bulk data. The embedded heatmap widget is available at https://github.com/ebi-gene-expression-group/atlas-heatmap (DOI: 10.5281/zenodo.17401749) and the single-cell tertiary analysis workflow at https://github.com/ebi-gene-expression-group/scxa-tertiary-workflow (DOI: 10.5281/zenodo.17401767). The ExpressionAtlas R/Bioconductor package (https://bioconductor.org/packages/ExpressionAtlas/) provides programmatic access to the resource. All relevant information about data processing and file access is provided through the ‘Supplementary information’ or ‘Download’ tabs on each experiment page. These tabs link directly to the corresponding folder on the Expression Atlas FTP site (https://ftp.ebi.ac.uk/pub/databases/microarray/data/atlas/experiments/) as well as to the archives from which the raw data (ENA [54] or EGA) and metadata (ArrayExpress or GEO) were obtained.
