# Supplementary material for: Expression Atlas in 2026: enabling FAIR and open expression data through community collaboration and integration
Source: Nucleic Acids Res. 2025 Dec 10;54(D1):D147–57. doi: 10.1093/nar/gkaf1238 (PMC12807774; doi:10.1093/nar/gkaf1238)
Supplement: gkaf1238_Supplemental_File [file gkaf1238_supplemental_file.pdf]

Supp. Table S1: Gramene plant species represented in Expression Atlas with number of studies by baseline and differential expression

Plant Species	Baseline	Differential	Total Bulk Expression Studies
Arabidopsis thaliana	18	615	633
Oryza sativa	15	98	113
Zea mays	33	58	91
Vitis vinifera	10	23	33
Glycine max	9	13	22
Solanum lycopersicum	5	15	20
Triticum aestivum	5	13	18
Hordeum vulgare	3	13	16
Sorghum bicolor	11	5	16
Medicago truncatula	0	10	10
Solanum tuberosum	1	7	8
Brachypodium distachyon	2	5	7
Populus trichocarpa	1	5	6
Brassica rapa	2	3	5
Chlamydomonas reinhardtii	1	3	4
Physcomitrella patens	0	4	4
Musa acuminata	1	2	3
Brassica napus	1	2	3
Theobroma cacao	1	2	3
Beta vulgaris	1	1	2
Arabidopsis lyrata	1	1	2
Brassica oleracea	1	1	2
Aegilops tauschii	1	0	1
Setaria italica	1	0	1
Triticum urartu	0	1	1
Trifolium pratense	1	0	1
Prunus persica	0	1	1
Total	125	901	1026

Supp. Table S2: Gramene plant species represented in Single-cell Expression Atlas with number of studies

Plant Species	Total Single Cell Expression Studies
Arabidopsis thaliana	14
Oryza sativa	3
Solanum lycopersicum	2
Zea mays	2
Total	21
